# The indicative role of inflammatory index in the progression of periodontal attachment loss

**DOI:** 10.1186/s40001-023-01247-8

**Published:** 2023-08-17

**Authors:** Wenhao Zhang, Yulong Zhang, Cong Jin, Ruihan Fang, Ruixue Hua, Xiaodong Zang, Hengguo Zhang

**Affiliations:** 1 Key Laboratory of Oral Diseases Research of Anhui Province, College & Hospital of Stomatology, Anhui Medical University, Hefei, 230032 China; 2 The First Affiliated Hospital of USTC, Division of Life Sciences and Medicine, University of Science and Technology of China, Hefei, 230032 China; 3 Department of Dental Implantology, College & Hospital of Stomatology, Anhui Medical University, Hefei, 230032 China

**Keywords:** Periodontal attachment loss, Immune cell, NHANES, WBC, Neutrophils

## Abstract

**Objective:**

To explore the forewarning immunological indicators during periodontal attachment loss progression in American adults.

**Methods:**

A total of 5744 participants with periodontal attachment loss were enrolled from the National Health and Nutrition Examination Surveys (NHANES) 2009–2014. In which, dependent variable was the counts of teeth with severe attachment loss (depth of periodontal probing was above 5 mm). Independent variables were circulatory immunological indexes, including counts of white blood cells (WBC), Lymphocytes, Monocytes, Neutrophils, Eosinophils, and Basophils. The association among variables was examined using multivariable linear regression models, fitting with smoothing curves, and generalizing additive models.

**Results:**

Based on the indicators of 5744 subjects, we found that severe attachment loss tended to occur in the elderly or males and was accompanied by higher WBC, Monocytes, and Neutrophils, as well as lower poverty-income ratio and educational qualification. WBC (above the inflection point: 6200 cells/µL) and Neutrophils (above the inflection point: 3300 cells/µL) counts were positively associated with attachment loss progression in each multivariable linear regression model. On subgroup analyses, stratified by sex and race, the positive correlation of WBC or Neutrophils with severe attachment loss was stable in both men and women, as well as in all races except blacks (WBC β = − 0.0576, 95% CI − 0.1945 to 0.0793, Neutrophils β = − 0.0527, 95% CI − 0.2285 to 0.1231).

**Conclusion:**

Increasing WBC (above 6200 cells/µL) and Neutrophils (above 3300 cells/µL) counts were risk indicators of severe periodontal attachment loss among all races, except in blacks.

## Introduction

With a prevalence of 47% in the American population over 30 years old [1, 2], periodontitis impaired the aesthetic and oral function [3, 4], mainly manifested as attachment loss and alveolar bone resorption [5–7]. Current studies focused on the correlated inflammation or immunity factors of severe periodontitis [8], but the in-depth monitoring of immunological indicators during attachment loss progression remains unknown.

On accounts of the abundant systemic circulation of the maxillofacial region, periodontitis causes or exacerbates comorbidities and resulting changes in circulating immune indicators (including white blood cells, Neutrophils, etc.) [7, 9, 10]. Previous studies showed that severe periodontitis was accompanied by changes in the levels of inflammatory indicators [11, 12], for example, patients with periodontitis are often accompanied by an increase in mean platelet volume and a decrease in eosinophilic counts. Meanwhile, circulatory immune indexes were widely utilized as forewarning or diagnostic criteria of infectious diseases. For instance, white blood cell (WBC) counts were generally expected to be closely associated with a respiratory infection, which diagnoses pneumonia, determines its etiology, and predict disease progression [13, 14]. To forewarn the occurrence and progression of severe periodontitis, it is necessary to explore the potential relationship between circulatory immunological indexes and periodontal attachment loss progression.

In this study, we aimed to explore the association between severe attachment loss progression and circulatory immunological indicators by multivariable linear regression based on the NHANES database. Severe periodontal attachment loss tended to occur in males and was accompanied by higher WBC, Monocytes, and Neutrophils. Furthermore, WBC, Monocytes, and Neutrophils counts were closely associated with periodontal attachment loss. This study sought to shed light on the correlation between the progression of severe periodontitis and immune-related indicators.

## Methods

### Study population

Data of the current study were abstracted from the National Health and Nutrition Examination Survey (NHANES). In total, 14,071 participants were enrolled from NHANES between 2009 to 2014. After excluding participants (n = 2336) with incomplete records of periodontal examination, 11,735 participants were available. Further, excluding participants who had the attachment loss < 5 mm or > 2 mm (n = 5771), and with missing data of the immune cell examination (n = 222), 5744 participants were finally enrolled in the study. The study was approved by the review board of the National Center for Health Statistics.

### Dependent variable

Periodontal attachment loss was identified by dental examiners, who were dentists (D.D.S./D.M.D.) licensed in at least one U.S. state. All oral health assessments occurred in a designated room at the mobile examination center (MEC). In this experiment, attachment loss ≥ 5 mm was defined as severe attachment loss [15]. In addition, dependent variable was counts of teeth with severe attachment loss.

### Independent variables and covariates

Circulatory immunological indexes (including white blood cells, Lymphocytes, Monocytes, Neutrophils, Eosinophils, and Basophils) were obtained from laboratory data. For covariates, including age, gender, education level (< 9th grade, 9–11th grade, high school, college, etc.), race/ethnicity (Mexican American, Hispanic, non-Hispanic White, non-Hispanic Black, and other races), PIR (poverty income ratio), Body Mass Index (BMI). Circulatory immunological indexes and other covariates acquisition processes are available on the NHANES dataset (http://cdc.gov/nchs/nhanes).

### Statistical analysis

All statistical analyses were performed by using R (http://www.R-project.org), with statistical significance set at P < 0.05. All estimates were calculated by using sample weights following the analytical guideline edited by NCHS. Three multivariable linear regression models were constructed by hierarchical multiple regression to test the significance of variables: model 1, no covariates were adjusted; model 2, age, gender, and race were adjusted; model 3, all covariates presented in Table 1 were adjusted. Furthermore, smooth curve fittings and generalized additive models were used to address the nonlinearity. The inflection points were calculated using a recursive algorithm, with a two-piecewise linear regression model conducted on both sides of the inflection point when nonlinearity was detected.

**Table 1:** Characteristics of the study population

Periodontal attachment loss progression	Q1 (n = 1075)	Q2 (n = 1207)	Q3 (n = 1920)	Q4 (n = 1542)	P value
Age (years)	41.9429 ± 10.3654	53.8780 ± 14.2286	57.0781 ± 13.5825	57.3122 ± 12.1847	< 0.000001
Gender (%)					< 0.000001
Male	46.5866	55.4821	59.5286	71.3919	
Female	53.4134	44.5179	40.4714	28.6081	
Race (%)					< 0.000001
Mexican American	10.362	15.0822	15.9375	19.7697	
Other Hispanics	7.6338	10.9928	10.1701	8.6655	
Non-Hispanic whites	57.3723	40.5504	39.5653	33.106	
Non-Hispanic blacks	12.6704	23.9272	25.0664	28.4604	
Other races	11.9614	9.4473	9.2608	9.9984	
BMI (kg/m^2^)	30.2613 ± 7.2195	31.1544 ± 7.1552	30.9483 ± 7.5338	30.1925 ± 7.2611	0.000479
PIR	3.4271 ± 1.5579	2.5616 ± 1.5997	2.3715 ± 1.5809	2.0498 ± 1.4968	< 0.000001
Education (%)					< 0.000001
Below Grade 9	3.5378	9.6521	11.6025	15.0298	
Grades 9–11	4.7052	14.8045	16.3671	19.18	
High school graduate	13.4946	23.3908	24.5514	28.3724	
College /AA degree	30.885	27.3026	28.0989	24.436	
College degree or above	47.1936	24.7337	18.8105	12.5115	
Refused to answer	0.1034	0.1164	0.196	0.4702	
Don't know	0.0805	0.0000	0.3737	0.0000	
Circulatory immune cell counts (1000 cells/µL)					
WBC	7.0093 ± 2.0269	7.1159 ± 2.0240	7.2347 ± 2.1349	7.4104 ± 2.2168	0.00001
Monocyte	0.5189 ± 0.1674	0.5415 ± 0.1893	0.5556 ± 0.2220	0.5748 ± 0.1992	< 0.000001
Neutrophils	4.1326 ± 1.6089	4.1839 ± 1.5710	4.2905 ± 1.7428	4.4441 ± 1.7823	0.000008
Eosinophils	0.1924 ± 0.1837	0.2097 ± 0.1729	0.2124 ± 0.1793	0.2088 ± 0.1666	0.051553
Basophils	0.0390 ± 0.0536	0.0441 ± 0.0626	0.0444 ± 0.0612	0.0440 ± 0.0600	0.088395
Lymphocyte	2.1183 ± 0.6694	2.1346 ± 0.7644	2.1286 ± 0.7263	2.1345 ± 0.7398	0.942726

Data are presented as n (%), and mean (SD)

PIR, poverty income ratio; BMI, body mass index; WBC, White blood cell counts

## Results

The demographic and laboratory data of the 5744 participants (3217 men and 2527 women), with the weighted characteristics of the participants subclassified based on quartiles of attachment loss teeth counts (Q1: 0; Q2: 1; Q3: 2–5; and Q4: 6–27), as presented in Table 1. There were significant differences in baseline characteristics between the attachment loss teeth quartiles, except for the counts of Eosinophils, Basophils, and Lymphocytes. Participants with generalized periodontal attachment loss tend to occur in the elderly or males and are accompanied with higher BMI, WBC, Monocytes, Neutrophils, and Eosinophils, as well as lower PIR and educational qualification.

The results of the multivariate regression analyses are presented in Tables 2 and 3. In the unadjusted model, WBC (β = 0.1632, 95% CI 0.1051–0.2214, P < 0.000001) and Neutrophils (β = 0.2069, 95% CI 0.1344–0.2795, P < 0.000001) were positively correlated to attachment loss progression. After adjustment for confounders, those positive associations were stable in model 2 (P < 0.000001) and model 3 (P < 0.000001). After converting WBC and Neutrophils from continuous variables to categorical variables (quartiles), the trends remained significant among different WBC or Neutrophils quartile groups (P < 0.001). Furthermore, individuals in the highest WBC and Neutrophils quartile had 0.8392 (P < 0.00001) and 0.8663 (P < 0.00001) more attachment loss than those in the lowest quartile, respectively.

**Table 2:** Association between WBC and periodontal attachment loss progression

	Model 1, β (95% CI) P value	Model 2, β (95% CI) P value	Model 3, β (95% CI) P value
WBC	0.1632 (0.1051, 0.2214) < 0.000001	0.2521 (0.1958, 0.3083) < 0.000001	0.1889 (0.1289, 0.2489) < 0.000001
Quintiles of WBC			
Q1	Reference	Reference	Reference
Q2	− 0.2316 (− 0.5897, 0.1265) 0.204932	− 0.0003 (− 0.3442, 0.3435) 0.998490	− 0.0172 (− 0.3730, 0.3386) 0.924533
Q3	0.0973 (− 0.2642, 0.4589) 0.597791	0.4147 (0.0653, 0.7641) 0.020044	0.2829 (− 0.0815, 0.6474) 0.128185
Q4	0.6330 (0.2751, 0.9909) 0.000531	1.1660 (0.8183, 1.5137) < 0.000001	0.8392 (0.4706, 1.2078) 0.000008
P for trend	< 0.001	< 0.001	< 0.001
Stratified by gender			
Male	0.1920 (0.1065, 0.2776) 0.000011	0.2726 (0.1886, 0.3566) < 0.000001	0.2123 (0.1235, 0.3012) 0.000003
Female	0.1553 (0.0841, 0.2264) 0.000020	0.2255 (0.1554, 0.2957) < 0.000001	0.1399 (0.0649, 0.2149) 0.000262
Stratified by race			
Mexican American	0.2254 (0.0562, 0.3946) 0.009180	0.2229 (0.0604, 0.3855) 0.007308	0.2211 (0.0416, 0.4006) 0.015987
Other Hispanics	0.3352 (0.1662, 0.5043) 0.000114	0.3612 (0.2001, 0.5222) 0.000013	0.2687 (0.0889, 0.4486) 0.003570
Non-Hispanic whites	0.3138 (0.2334, 0.3942) < 0.000001	0.3612 (0.2834, 0.4390) < 0.000001	0.2795 (0.1976, 0.3614) < 0.000001
Non-Hispanic blacks	− 0.0792 (− 0.2108, 0.0523) 0.237836	− 0.0051 (− 0.1336, 0.1233) 0.937387	− 0.0576 (− 0.1945, 0.0793) 0.409560
Other races	0.2240 (0.0212, 0.4268) 0.030742	0.3167 (0.1234, 0.5100) 0.001384	0.2674 (0.0573, 0.4776) 0.012921

Model 1, no covariates were adjusted

Model 2, age, gender, and race were adjusted

Model 3, all covariates presented in Table 1 were adjusted

**Table 3:** Association between Neutrophils and periodontal attachment loss

	Model 1, β (95% CI) P value	Model 2, β (95% CI) P value	Model 3, β (95% CI) P value
Neutrophils	0.2069 (0.1344, 0.2795) < 0.000001	0.3066 (0.2359, 0.3773) < 0.000001	0.2347 (0.1598, 0.3097) < 0.000001
Quintiles of Neutrophils			
Q1	Reference	Reference	Reference
Q2	− 0.2085 (− 0.5631, 0.1460) 0.249030	− 0.0064 (− 0.3484, 0.3357) 0.970927	− 0.0122 (− 0.3669, 0.3426) 0.946458
Q3	0.1752 (− 0.1782, 0.5285) 0.331213	0.4011 (0.0587, 0.7435) 0.021695	0.3051 (− 0.0516, 0.6618) 0.093678
Q4	0.7262 (0.3785, 1.0740) 0.000043	1.1668 (0.8267, 1.5068) < 0.000001	0.8663 (0.5065, 1.2260) 0.000002
P for trend	< 0.001	< 0.001	< 0.001
Stratified by gender			
Male	0.2543 (0.1476, 0.3610) 0.000003	0.3392 (0.2337, 0.4447) < 0.000001	0.2728 (0.1615, 0.3842) 0.000002
Female	0.1684 (0.0794, 0.2573) 0.000211	0.2617 (0.1733, 0.3500) < 0.000001	0.1583 (0.0645, 0.2521) 0.000957
Stratified by race			
Mexican American	0.3221 (0.1109, 0.5334) 0.002878	0.2908 (0.0874, 0.4942) 0.005188	0.3080 (0.0846, 0.5314) 0.007031
Other Hispanics	0.3907 (0.1801, 0.6013) 0.000303	0.3882 (0.1872, 0.5893) 0.000170	0.3012 (0.0747, 0.5277) 0.009441
Non-Hispanic whites	0.3797 (0.2799, 0.4795) < 0.000001	0.4078 (0.3114, 0.5043) < 0.000001	0.3136 (0.2131, 0.4142) < 0.000001
Non-Hispanic blacks	− 0.0486 (− 0.2192, 0.1220) 0.576815	− 0.0120 (− 0.1536, 0.1776) 0.887232	− 0.0527 (− 0.2285, 0.1231) 0.556745
Other races	0.3877 (0.1270, 0.6483) 0.003673	0.4556 (0.2079, 0.7032) 0.000335	0.4050 (0.1333, 0.6767) 0.003622

Model 1, no covariates were adjusted

Model 2, age, gender, and race were adjusted

Model 3, all covariates presented in Table 1 were adjusted

**Fig. 1:**
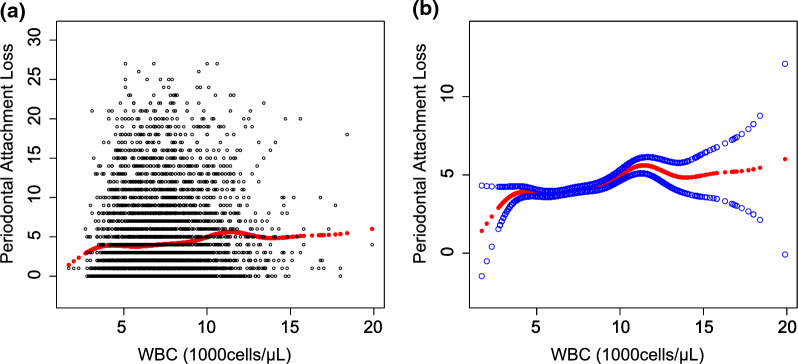
The association between WBC and periodontal attachment loss. **a** Each black point represents a sample. **b** Solid rad line represents the smooth curve fit between variables. Blue bands represent the 95% of confidence interval from the fit

**Fig. 2:**
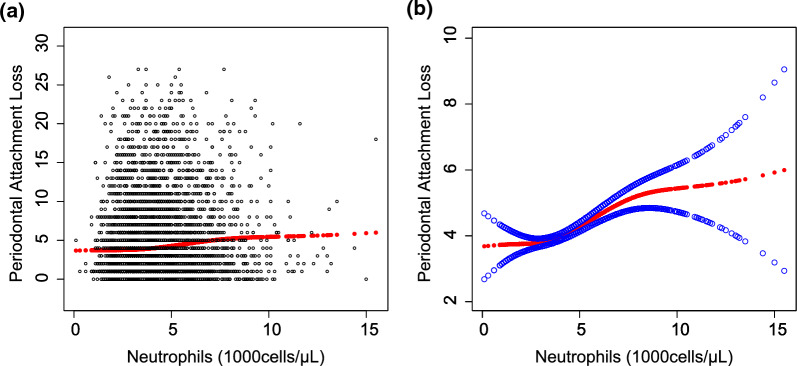
The association between Neutrophils and periodontal attachment loss. **a** Each black point represents a sample. **b** Solid rad line represents the smooth curve fit between variables. Blue bands represent the 95% of confidence interval from the fit

Smooth curve fittings and generalized additive models used to characterize the nonlinear relationship between WBC/Neutrophils and attachment loss progression are shown in Figs. 1, 2. The points of inflection were identified using the two-piecewise linear regression models, at 6200 cells/µL (WBC counts) and 3300 cells/µL (Neutrophils counts), respectively (Tables 4 and 5). For WBC < 620 cells/µL or Neutrophils < 3300 cells/µL, every 1000 cells/µL increase of WBC or Neutrophils was correlated with 0.0784 (95% CI −0.2836 to 0.1269) or 0.1494 (95% CI − 0.4550 to 0.1562) less attachment loss; by comparison, for individuals with WBC > 6200 cells/µL or Neutrophils > 3300 cells/µL, every 1000 cells/µL increase of WBC or Neutrophils was significantly associated with 0.2273 (95% CI 0.1492–0.3054) or 0.2737 (95% CI 0.1823–0.3651) more attachment loss.

**Table 4:** Threshold effect analysis of WBC on the progression of attachment loss using the two piecewise linear regression model

The number of caries	Adjusted β (95% CI), P value
Fitting by the standard linear model	0.1632 (0.1051, 0.2214) < 0.0001
Fitting by the two-piecewise linear model	
Inflection point	6.2
WBC < 6.2 (1000 cells/µL)	− 0.0784 (− 0.2836, 0.1269) 0.4542
WBC > 6.2 (1000 cells/µL)	0.2273 (0.1492, 0.3054) < 0.0001
Log likelihood ratio	0.016

**Table 5:** Threshold effect analysis of Neutrophils on the progression of attachment loss using the two piecewise linear regression model

The number of caries	Adjusted β (95% CI), P value
Fitting by the standard linear model	0.2069 (0.1344, 0.2795) < 0.0001
Fitting by the two-piecewise linear model	
Inflection point	3.3
Neutrophils < 3.3 (1000 cells/µL)	− 0.1494 (− 0.4550, 0.1562) 0.3379
Neutrophils > 3.3 (1000 cells/µL)	0.2737 (0.1823, 0.3651) < 0.0001
Log likelihood ratio	0.019

**Fig. 3:**
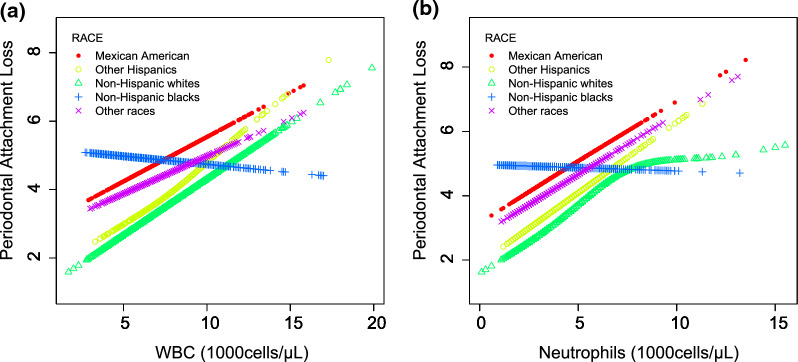
The association between WBC, Neutrophils and periodontal attachment loss, stratified by race

On subgroup analyses, stratified by sex and race/ethnicity, reported in Tables 2 and 3, the positive correlation among WBC, Neutrophils and attachment loss progression remained in both males (P < 0.0001) and females (P < 0.001), as well as in Mexican American (P < 0.05), Hispanics (P < 0.05), whites (P < 0.000001), and other races (P < 0.05), but not in blacks. Among blacks, WBC (β = -0.0576, 95%CI: -0.1945–0.0793) or Neutrophils (β =  − 0.0527, 95% CI − 0.2285 to 0.1231) were negatively associated with attachment loss progression (Fig. 3).

## Discussion

Accompanied with attachment loss, severe periodontitis caused serious aesthetic problems and reduced chewing efficiency [16, 17]. Previous studies suggested that the progression of attachment loss was accompanied with increasing circulatory immunological indexes in severe periodontitis [13, 14], but immune cell counts serve as forewarning or diagnostic criteria for severe attachment loss or periodontitis progression remains unclear. Our multivariate logistic regression analyses indicated that elevated WBC or Neutrophils was correlated with attachment loss progression. However, on subgroup analysis, we identified the negative relationship between WBC or Neutrophils and attachment loss among blacks.

Phagocytosis by WBC, Neutrophils, and Monocytes constitutes the main defense mechanism against bacterial challenges in periodontitis [18]. As the enriched immune cell killing bacteria and destroying inflamed tissues [19], immunity homeostasis was essential for periodontal attachment maintenance [20]. In consist with our results, a previous study showed that severe periodontitis accompanied with higher WBC or Neutrophils levels than the healthy population [6]. On the other hand, infectious viruses, including Human simplex virus-1, Epstein-Barr virus, and Human cytomegalo virus, were commonly involved in the occurrence and development of periodontitis or attachment loss [21]. Simple viral infections accompanied with the decrease of WBC and Neutrophils [22], while combination of bacterial infections promotes WBC elevation [23]. Recent research identified the inflection point of fluctuating confounder was utilized as forewarning of periodontitis risk, and periodontal status [24]. Our multivariable linear regression analyses suggested that participants with WBC above 6200 cells/µl or Neutrophils above 3300 cells/µl obtained higher risk of periodontal attachment loss, which can serve for severe periodontitis prevention and treatment.

Previous studies identified the racial differences of WBC or Neutrophils counts between blacks and other races [25, 26]. In which, WBC or Neutrophils counts were significantly lower in blacks during dental plaque accumulation, but hyperactivity of circulating neutrophils appeared in blacks [26]. Importantly, the reactivity of oxidative burst was significantly lower in neutrophils from blacks, especially from African American males [27]. Periodontal attachment loss-related racial differences can provide accurate guidance for subsequent community prevention.

This study demonstrated the association between circulatory immunological indicators and periodontal attachment loss. However, several limitations are noticeable. First, all self-reported information might cause recall bias or misclassification bias. Second, further studies with larger sample sizes using more relevant variables (such as gingival sulcus fluid data and bone destruction) may discover more accurate factors in periodontal attachment loss. Third, circulating immune cells counts were affected by the whole-body state, periodontal pocket related immunological indexes may have better reproducibility.

## Conclusion

Our study suggested that increasing WBC (above 6200 cells/µL) and Neutrophils (above 3300 cells/µL) counts were potential risk indicators of periodontal attachment loss progression, which provides an early warning for severe periodontitis.

## Data Availability

Publicly available datasets were analyzed in this study. Data used for this study are available on the NHANES website: https://wwwn.cdc.gov/nchs/nhanes/.
